# Annotations

**Published:** 1901-08-10

**Authors:** 


### Annotations.

### Calisthenics for the Early Riser.

There are certain drawbacks to early rising. Without saying, with the sluggard, that early rising puffs men up with pride, one may at least admit that a habit which leads a man to roam the house at a time when it is usually given over to the housemaids is an inconvenient one. What to do when one has got up and where to go before breakfast on a wet morning are questions which must be faced. Dr. Lillian Powell, writing in the *Humanitarian* on "The Physical Culture of Girls," suggests just what is wanted. Every girl, she says, ought to practice calisthenics in her bedroom after her bath. Parallel bars or a trapeze can be put up in every bedroom, and even without such apparatus many useful exercises can be gone through which will go far in effecting the same purpose, and when the girl is "grown up" and has become a woman, she should continue the same course. Every morning she should devote half an hour to calisthenics, prolonging rather than shortening the time as she grows older. If possible, Dr. Powell would have her do the same thing again before going to bed; it is, however, as an occupation for the early riser, a means of usefully filling up the time, between getting up and coming down, that we urge the advantages of this practice for which Dr. Lillian Powell promises a great reward. With delightful optimism she says: "As to age, if a woman does her calisthenics one day she can do them the next, and so on *ad infinitum.* It is only when she begins to neglect them that, from want of practice, stiffness and flabby superfluous flesh come to prevent freedom of movement and beauty of form." And if woman, why not man? In the evolution of the race we have attained the morning bath; now we must aim at the morning calisthenics. Instead of yawning, we shall then come down hungry; instead of growing stodgy with advancing age, we shall keep our figures. Of course there are dumbbells, but these do not entirely meet the case. What is wanted is such movement that at least once a day every muscle shall be exercised and every joint moved to its full extreme. Whether to take the bath after or before is another question. Unless the weather were so cold as to make exercise necessary to produce reaction, we should take the bath after rather than before.

### Cold Storage in Public Abattoirs.

The present is not the moment, perhaps, at which to enter upon the discussion which has been going on for some time past as to the desirability of erecting public abattoirs in all large towns and entirely prohibiting the use of the private slaughter houses the continued employment of which constitutes the main standing difficulty in the way of providing for the proper and complete inspection of meat. We would however point out how very largely the position of this question, and the standpoint from which it has to be regarded by all parties, is altered by the developments which have of late years taken place in the system of cold storage. When the question first became a prominent one, the abolition of private slaughter houses was regarded by the butchers as pure confiscation, and we are not sure that even now there are not some who look at it in this light. But the modern public abattoir with its cold storage chambers puts the matter in a different light, for it tends to relieve the butcher of what has always been one of the chief drawbacks of his trade, namely, the liability to loss from the spoiling of his meat in case of unfavourable weather or unexpected oscillations of the market. With cold chambers meat practically ceases to be a perishable article, and thus any measure which tends to drive the slaughtering of cattle to the public abattoirs, so far from robbing the butcher of a valuable asset offers him a definite advantage in the conduct of his business. Mr. Shirley Murphy, medical officer to the County of London, has lately stated that all the public slaughter houses in Germany which were visited by the members of the Tuberculosis Commission, were found to be self supporting, and he gives ample reasons for believing that not only would the introduction of such establishments in our large towns, and their compulsory use, be of great advantage to the community, but it would actually be a benefit to the butchers themselves, by removing from their trade much of the uncertainty and irregularity with which it is now surrounded

### Dispensing.

From the far North comes a lesson in the proprieties of medical conduct. The graduates of the University of Aberdeen are officially informed that the Senatus Academicus has passed the following resolution:—

> Whilst it is admitted that the exigencies of practice may sometimes render it unavoidable for a medical practitioner to supply to his patients the remedies which he prescribes, the Senatus Academicus of this University is of opinion that it is undesirable, and detrimental to the position of medical graduates of the University, that this custom should be followed under other circumstances; and, further, it regards the sale of objects other than remedies by its medical graduates as under all circumstances to be strongly deprecated.

A similar resolution has, we are told, been adopted by the University of Edinburgh. With the last paragraph of this resolution the great majority of self-respecting practitioners will, we think, thoroughly agree. It puts in definite shape the opinion, which has for long been generally held, that the keeping of a shop is entirely foreign to the work of the qualified medical practitioner. But when these two Universities issue a general pronouncement like this against their graduates supplying to their own patients the remedies which they themselves have prescribed, except under such exceptional circumstances as may "sometimes" render such a course "unavoidable," we may fairly inquire whether the facts of life are such as to make it at all likely that these gentlemen, who, if we are to believe common report, flood the medical market, will restrict themselves to pure prescribing, and will cease to compete with other qualified practitioners for clubs and various forms of contract service, the very essence of which generally is that they shall supply "attendance and medicine" at so much a head. We can only describe this pretty little resolution as a bit of bounce on the part of these two Universities; a simple and easy method of declaring that their graduates are not as other graduates —dispensers, drug-sellers, and dealers in tooth-brushes, nor even as these licentiates of the Royal Colleges. After all, the question of dispensing will have to be decided according to the convenience of the public rather than by any resolutions of any Senatus Academicus. There is nothing any more derogatory to his position in the general practitioner making up his own medicines than there is in the surgeon doing his own operation, or in the artist setting his own palette; and certainly there is nothing in it half so ignominious as there is in the various investigations of the secretions which the modern physician is bound to undertake if he would search out the secrets of disease. The fact that he does not dispense medicines gives no man the right to hold himself superior to the one who does. Position in the profession fortunately still depends on honesty and skill, not on obedience to fanciful academic rules.

